# Work activities after 3-year follow-up in ICU patients with traumatic brain injury

**DOI:** 10.1186/cc13670

**Published:** 2014-03-17

**Authors:** M Arias-Verdu, M Delange Van der Kroft, E Curiel-Balsera, A Muñoz-Lopez, J Fernandez-Ortega, R Rivera-Fernandez, M Prieto-Palomino

**Affiliations:** 1 Carlos Haya University Hospital, Malaga, Spain; 2 County Hospital of Axarquia, Velez Malaga, Spain

## Introduction

The aim was to study work activities after 3 years in patients admitted to the ICU with traumatic brain injury (TBI).

## Methods

A prospective cohort study in an ICU of a reference hospital. We studied ICU-admitted patients consecutively with TBI between 2004 and 2008. We used validated scales including the Glasgow Outcome Scale (GOS) and the Project for the Epidemiological Analysis of Critical Care Patients Quality of Life questionnaire.

## Results

We studied 531 patients, age 40.35 ± 19.75 years, APACHE II 17.94 ± 6.97 points, Glasgow Coma Scale at admission 7.53 ± 3.83 points. Cranial tomography at admission was: diffuse injury type I (10.4%), diffuse injury type II (28.1%), diffuse injury type III (24.5%), diffuse injury type IV (8.3%), evacuated mass lesion (22.6%), non-evacuated mass lesion (6.2%). Hospital mortality was 28.6%, 171 (32.2%) patients died after 1 year (6.6% missing) and 181 (34.1%) died after 3 years (16.2% missing). Regarding work activities, after 1 year, 28.5% of 326 patients evaluated have no difficulties with work, 4.6% have difficulties but work as before, 10.1% work only part-time or have changed to a job requiring minimum effort and 56.7% of patients do not work. After 3 years, 41.2% of 238 patients evaluated have no difficulties with work, 4.6% have difficulties but work as before, 12.6% work only part-time or have changed to a job requiring minimum effort and 41.6% of patients do not work. Evolution between 1 and 3 years by the McNemar test was statistically significant (*P *< 0.001). A total of 173 patients were in similar situation, only one had deteriorated and in 62 (26.05%) patients the evaluation of work activity had improved.

## Conclusion

After 1 year of admission from the ICU with TBI, more than 50% of patients have difficulties with work. After 3 years the number of patients who work has increased although approximately 40% of the surviving patients do not work.

